# *OTC* intron 4 variations mediate pathogenic splicing patterns caused by the c.386G>A mutation in humans and spf^ash^ mice, and govern susceptibility to RNA-based therapies

**DOI:** 10.1186/s10020-021-00418-9

**Published:** 2021-12-14

**Authors:** Claudia Sacchetto, Laura Peretto, Francisco Baralle, Iva Maestri, Francesca Tassi, Francesco Bernardi, Stan F. J. van de Graaf, Franco Pagani, Mirko Pinotti, Dario Balestra

**Affiliations:** 1 Department of Life Sciences and Biotechnology, University of Ferrara, Via Fossato di Mortara 74, 44121 Ferrara, Italy; 2 Department of Molecular Genetics, University of Maastricht, Maastricht, The Netherlands; 3 Liver Research Center, Q AREA Science Park, Trieste, Italy; 4 Department of Translational Medicine and for Romagna, Pathology Unit of Pathologic Anatomy, Histology and Cytology, University of Ferrara, Ferrara, Italy; 5 Tytgat Institute for Liver and Intestinal Research, Amsterdam Gastroenterology Endocrinology and Metabolism, Academic Medical Center, Amsterdam, The Netherlands; 6 Human Molecular Genetics, ICGEB - International Center for Genetic Engineering and Biotechnology, Trieste, Italy

**Keywords:** OTC deficiency, Pathogenic mRNA splicing, Spf^ash^ mouse model, Nucleotide variations, U1snRNA

## Abstract

**Background:**

Aberrant splicing is a common outcome in the presence of exonic or intronic variants that might hamper the intricate network of interactions defining an exon in a specific gene context. Therefore, the evaluation of the functional, and potentially pathological, role of nucleotide changes remains one of the major challenges in the modern genomic era. This aspect has also to be taken into account during the pre-clinical evaluation of innovative therapeutic approaches in animal models of human diseases. This is of particular relevance when developing therapeutics acting on splicing, an intriguing and expanding research area for several disorders. Here, we addressed *species*-specific splicing mechanisms triggered by the OTC c.386G>A mutation, relatively frequent in humans, leading to Ornithine TransCarbamylase Deficiency (OTCD) in patients and spf^ash^ mice, and its differential susceptibility to RNA therapeutics based on engineered U1snRNA.

**Methods:**

Creation and co-expression of engineered U1snRNAs with human and mouse minigenes, either wild-type or harbouring different nucleotide changes, in human (HepG2) and mouse (Hepa1-6) hepatoma cells followed by analysis of splicing pattern. RNA pulldown studies to evaluate binding of specific splicing factors.

**Results:**

Comparative nucleotide analysis suggested a role for the intronic +10-11 nucleotides, and pull-down assays showed that they confer preferential binding to the TIA1 splicing factor in the mouse context, where TIA1 overexpression further increases correct splicing. Consistently, the splicing profile of the human minigene with mouse +10-11 nucleotides overlapped that of mouse minigene, and restored responsiveness to TIA1 overexpression and to compensatory U1snRNA. Swapping the human +10-11 nucleotides into the mouse context had opposite effects.

Moreover, the interplay between the authentic and the adjacent cryptic 5′ss in the human OTC dictates pathogenic mechanisms of several OTCD-causing 5′ss mutations, and only the c.386+5G>A change, abrogating the cryptic 5′ss, was rescuable by engineered U1snRNA.

**Conclusions:**

Subtle intronic variations explain species-specific *OTC* splicing patterns driven by the c.386G>A mutation, and the responsiveness to engineered U1snRNAs, which suggests careful elucidation of molecular mechanisms before proposing translation of tailored therapeutics from animal models to humans.

**Supplementary Information:**

The online version contains supplementary material available at 10.1186/s10020-021-00418-9.

## Background

Among the several mechanisms contributing to the complexity of the metazoan transcriptome, pre-mRNA splicing plays a central role (Nilsen and Graveley 2010). A pre-mRNA can undergo splicing through different pathways (alternative splicing; AS), which increases the variety of transcripts and potential protein isoforms, with pathophysiological and evolutionary implications (Merkin et al. 2012). Consistently, as the evolutionary distance from primates increases, the number of AS events decreases (Kim et al. 2007; Barbosa-Morais et al. 2012). Most AS is guided by cis-specific regulatory elements, located within exons and introns (Baralle and Baralle 2018). While the evolution of nucleotide variations can be evaluated by comparative genomic and RNAseq analyses, the study of their functional relevance across species, represents a major issue. This aspect, hardly predictable and so far poorly investigated, has to be taken into account also when exploring RNA therapeutics in animal models in the attempt to translate pre-clinical data into humans. RNA therapeutics consist of the viral or lipid-nanoparticle mediated delivery of engineered RNA to treat or prevent diseases, and has led to recent breakthrough innovations (Polack et al. 2020). Among molecules acting at RNA levels, engineered variants of the U1snRNA, the RNA component of the spliceosomal U1 ribonucleoprotein (U1snRNP) mediating 5′ss recognition and thus exon definition in the earliest splicing steps (De Conti et al. 2013), have proven to be effective in modulating splicing for therapeutic purposes. In particular, U1snRNA variants with increased complementarity with the 5′ss of the defective exon (named compensatory U1snRNA), or targeting the downstream intronic sequences (named Exon specific U1snRNA, ExSpeU1), have demonstrated their ability to rescue exon skipping caused by different types of mutations in cellular and animal models of several human diseases (Donadon et al. 2018, 2019; Scalet et al. 2018, 2019; Yamazaki et al. 2018; Balestra et al. 2019a, 2020a; Lee et al. 2019; Donegà et al. 2020; Martín et al. 2021).

A paradigmatic example is provided in Ornithine TransCarbamylase (OTC) deficiency (OMIM 311250), the most frequent (1:40.000–70.000) disease of the urea cycle in humans. The spf^ash^ mouse model carries the mutation c.386G>A; p.R129H (NP_000522) at the last position of *OTC* (NG_008471) exon 4 that has been also found in several patients with OTC deficiency (OTCD) (Tuchman et al. 2002; Yamaguchi et al. 2006; Rivera-Barahona et al. 2015). Studies in mice indicated that the p.R129H amino acid change does not impair OTC activity (Hodges and Rosenberg 1989; Zimmer et al. 1999), but the c.386G>A nucleotide change affects splicing. Noticeably, the degree of splicing impairment by the c.386G>A mutation substantially differs in humans and mice (Rivera-Barahona et al. 2015): in humans the pathogenic splicing pattern is characterized by exon 4 skipping and usage of a cryptic 5′ss at c.386+5 position, with only trace levels of correct transcripts; in mice the nucleotide change leads to exon 4 skipping and usage of an intronic cryptic 5′ splice site (ss) at c.386+49 position but it is also associated with appreciable levels of correct transcripts, accounting for hepatic OTC activity (5–10% of WT) and a mild phenotype.

These differences offer the opportunity for a deep investigation of the functional relevance of species-specific gene variations. Moreover, they could affect correction efficiency of U1snRNA variants designed to force exon 4 inclusion, which we showed to be capable to efficiently rescue OTC expression at both RNA and protein levels in the spf^ash^ mouse model (Balestra et al. 2020a).

Here, we dissected the molecular mechanisms underlying the *specie*-specific OTC exon 4 splicing pattern and demonstrated the key role of subtle intronic changes downstream of the authentic exon 4 5′ss, which also explain a differential responsiveness to RNA-based correction based on engineered U1snRNA.

## Methods

### Expression vectors and splicing assays

To create the human *OTC* minigene (OTC^h^), the genomic region of human *OTC* gene (NG_008471) including the last 535 bp of intron 3, exon 4 (88 bp) and the first 681 bp of intron 4 was amplified from the DNA of a healthy subject using high-fidelity *PfuI* DNA-Polymerase (ThermoFisher Scientific, Waltham, MA, USA) and primers hOTCF and hOTCR, and subsequently cloned into the pTB (41) by exploiting the *Nde*I restriction site inserted within primers. The mouse OTC minigene (OTC^m^) was available from previous studies (Balestra et al. 2020a). Nucleotide changes were introduced into the human and mouse OTC minigenes by mutagenesis (Balestra et al. 2020a). Expression vectors for the U1snRNA variants were created as previously reported (Balestra et al. 2015).

Human (HepG2) and mouse (Hepa1-6) hepatoma cells were cultured and seeded in 12-well plates and transiently transfected with one microgram of each expression vectors using Lipofectamine 2000 reagent (ThermoFisher Scientific, Waltham, MA, USA), as previously described (Ferraresi et al. 2020). Twenty-four hours post-transfection the total RNA was isolated with Trizol (ThermoFisher Scientific, Waltham, MA, USA), reverse transcribed and amplified with primers Alpha and Bra oligonucleotides designed on the upstream and downstream exons, respectively. The PCR was run for 40 cycles at the following conditions: 30 s at 95 °C, 30 s at 56 °C and 40 s at 72 °C. Amplicons were resolved on 2.5% agarose gel, and bands analyzed by densitometry through the UVITEC software (Cleaver Scientific, Warwickshire, UK). For denaturing capillary electrophoresis analysis, the fragments were fluorescently labeled by using primer Bra2 labeled with FAM and run on an ABI-3100 instrument, followed by analysis of peaks.

All constructs and transcript amplicons were validated by direct sequencing. Sequences of oligonucleotides are provided as Additional file 1: Tables S1 and S2.

All data reported are expressed as mean ± standard deviation (SD) and derive from at least three independent experiments.

### Computational analysis

Computational prediction of splice sites was conducted by using the Human Splicing Finder (http://www.umd.be/HSF/) tool.

The minimum free energy (MFE) of the interaction between the U1snRNA and donor splice site has been calculated by exploiting the RNAcofold Server (http://rna.tbi.univie.ac.at/cgi-bin/RNAWebSuite/RNAcofold.cgi) and using the 11-bp long sequence of 5′tail of U1snRNA and the targeted 5′ss as inputs.

### Pulldown assays

To affinity purify RNA-binding proteins, 100 pmol of 3′-biotin-coupled RNA oligonucleotides (Integrated DNA Technologies, Coralville, IA, USA) were incubated with 24 µg of HeLa nuclear extract (Ipracell, Mons, Belgium) and protease inhibitor cocktail (ThermoFisher Scientific, Waltham, MA, USA) in TENT buffer (10 mM Tris, 1 mM EDTA, 250 mM NaCl, 0.5% Triton X-100, pH 7.5) for 30 min at room temperature (20–25 °C). RNA–protein complexes were bound with 50 µg of streptavidin-coated magnetic beads (New England Biolabs, Ipswich, MA, USA) and washed with TENT buffer. Proteins were eluted with SDS sample buffer and analyzed by Western blotting with monoclonal mouse anti-TIA1 (0TI1D7, ThermoFisher Scientific, Waltham, MA, USA), polyoclonal mouse anti-Sam68 (PA5-62364, ThermoFisher Scientific, Waltham, MA, USA), monoclonal anti-GAPDH (ZG003, ThermoFisher Scientific, Waltham, MA, USA) and then with polyclonal goat anti-mouse HRP-conjugated (P0447, Dako, Jena, DE) antibodies. All RNA oligonucleotides are available in Additional file 1: Table S1.

## Results

### Nucleotide variations at +10-11 positions dictate species-specific splicing patterns

**Fig. 1:**
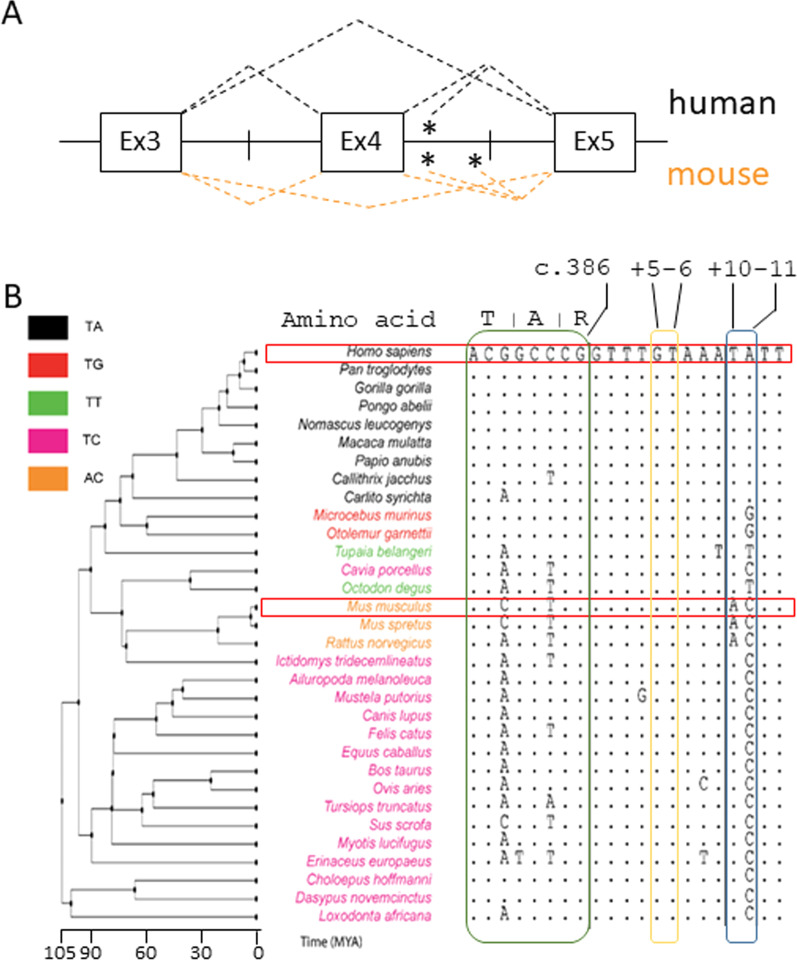
Species-specific splicing patterns and comparative sequence alignment of OTC exon 4 sequences. **A** Schematic representation of the OTC pre-mRNA focused on exon 4, with exons and introns indicated by boxes and lines, and of the alternative splicing events triggered by the c.386G>A mutation in the human and mouse context. Asterisks indicate cryptic 5′ss. **B** Multiple sequences alignment of the *OTC* exon 4 5′ss and the downstream region. The human sequence has been used as reference. Dots represents conserved nucleotide. The human and mouse sequences are included within red boxes. Exon 4 and nucleotides at position +10-11 are included within green and blue boxes, respectively. The cryptic 5′ss is highlighted by the yellow box. The phylogenetic tree, together with years (millions of years, MYA) of species separation, is reported on the left. Colors indicate the type of nucleotide couple at position +10-11

**Fig. 2:**
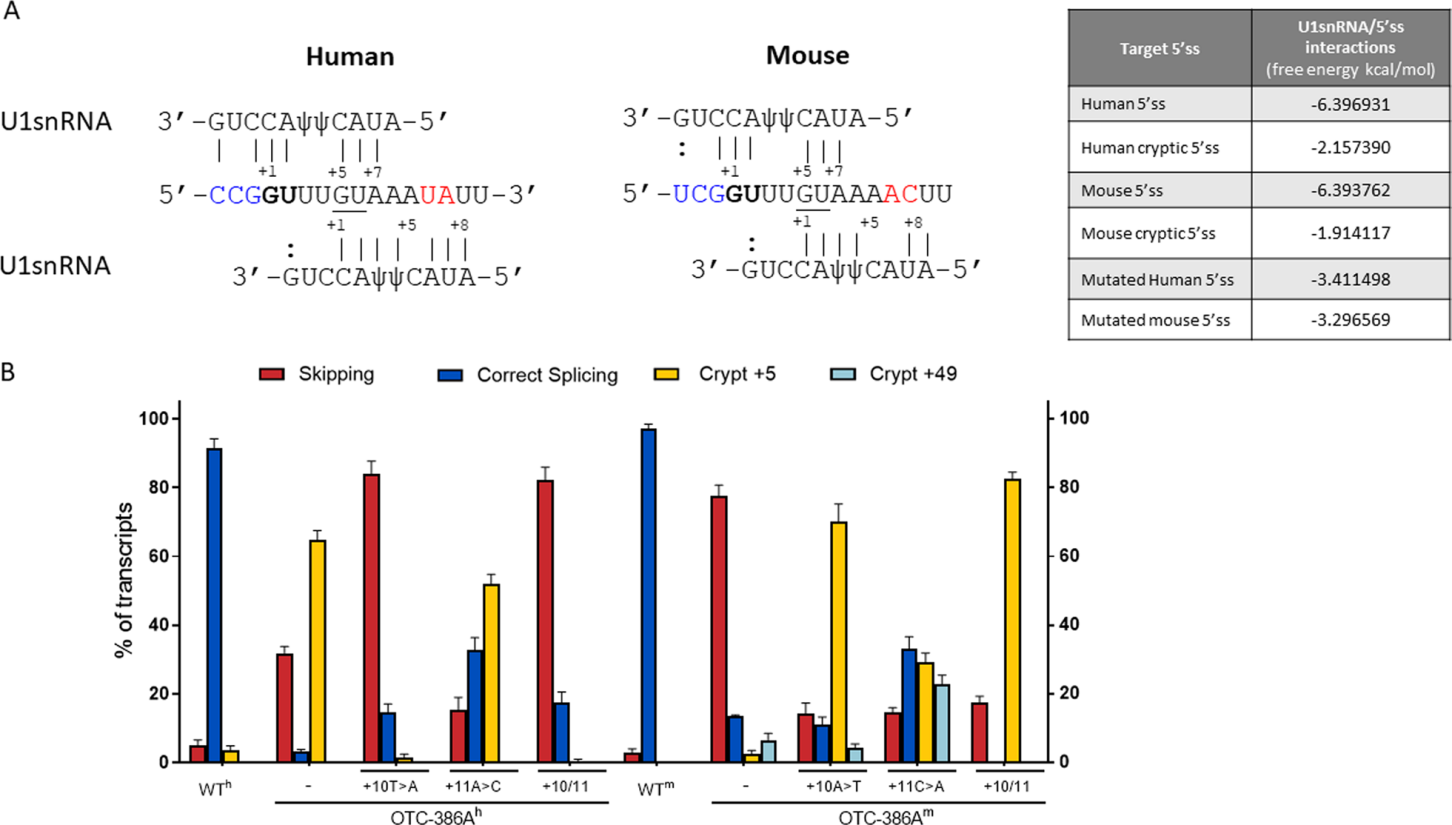
Nucleotide variations at +10-11 positions dictate species-specific splicing patterns. **A** Base pairing between the 5′ tail of the U1snRNA and the authentic (bold) or cryptic (underlined) 5′ss of human and mouse OTC pre-mRNA. Intronic nucleotides at position +10-11 are show in red. Continuous lines represent perfect matches while dashed line indicates partial complementarity. Nucleotides of OTC exon 4 are highlighted in blue. The minimum free energy (MFE) scores of interactions between the 5′ tail of U1snRNA and the targeted 5′ss are reported in the table on the right. **B** Splicing patterns of OTC^h^ and OTC^m^ minigenes, either wild-type (wt) or mutated, in HepG2 and Hepa1-6, respectively. Bar plots report the relative percentage of each transcript, expressed as mean ± standard deviation (SD) from three independent experiments. The transcript type is indicated on the top

To infer the mechanisms underlying the remarkably different splicing patterns triggered by the *OTC* c.386G>A mutation in humans and mouse (Fig. 1A) (Rivera-Barahona et al. 2015) we performed a sequence alignment of *OTC* exon 4 and the surrounding introns across species (Fig. 1B). Comparison of human and mouse sequences involving the authentic and the adjacent cryptic 5′ss reveals divergence at positions +10-11, corresponding to positions +6-7 of the cryptic 5′ss. In particular, nucleotide +10 is highly conserved among species, but in rodents, and adenine at +11 position characterizes primates. These variations are predicted to impact on the complementarity with the U1snRNA (Fig. 2A), which is higher in humans than in mice due to mismatches at positions +10-11.

Intrigued by these observations, we expressed human (h) and mouse (m) *OTC* hybrid minigenes in human (HepG2) and mouse (Hepa1-6) hepatoma cell lines, being OTC physiologically expressed in hepatocytes. To analyze splicing patterns, we exploited construct-specific primers to avoid confounding effects of endogenous OTC mRNA, followed by denaturing capillary electrophoresis to deeply evaluate all transcript forms. The c.386G>A mutation in OTC^h^ (OTC-386A^h^) induced exon skipping (32 ± 2% of all transcripts) and usage of the proximal (c.386+5) cryptic 5′ss (65 ± 3%), with trace levels of correct transcripts (3 ± 1%) (Fig. 2B). In the OTC^m^ (OTC-386A^m^), the mutation led to exon skipping (77 ± 3%), usage of the mouse-specific distal (c.386+49) cryptic 5′ss (7 ± 1%) and appreciable amount of correct transcripts (13 ± 1%). Interestingly, the capillary electrophoresis profile demonstrated for the first time the usage, albeit inefficient (2 ± 1.0%), of the proximal 5′ss even in the mouse context (Additional file 2: Fig. S1).

These data, which recapitulate and detail those previously reported, validated the experimental set-up and prompted us to define the role of +10-11 nucleotides on splicing patterns associated with the OTCD-causing mutation.

Mimicking the mouse context in the OTC-386A^h^ minigene by introducing the +10 T/A change abolished the usage of the cryptic 5′ss (from 65 ± 3% to 2 ± 1%, p < 0.0001) and ameliorated the usage of the authentic one (from 3 ± 1% to 14 ± 2%, p = 0.0017). Also, the +11A/C mouse-like change favored correct exon definition (33 ± 4%), and decreased the proportion of exon skipping (15 ± 4%). When these changes were combined, the OTC-386A^h^ minigene produced a splicing pattern that, considering the absence of the cryptic 5′ss at c.386+49 position, overlapped with that of the OTC-386A^m^ construct (Fig. 2B).

With the same mutagenesis-based approach, we swapped +10-11 nucleotides in the OTC-386A^m^ context. Either the human-inspired +10A/T or the +11C/A substitutions strongly favored the adjacent cryptic 5′ss (from 2 ± 1% to 70 ± 5% or 29 ± 2%, p < 0.0001, respectively) as compared to the authentic one. Accordingly, these changes in combination produced a splicing profile similar to the OTC-386A^h^ minigene, with virtually undetectable levels of correct transcripts.

Taken together these data indicate a key role of the +10-11 nucleotides in dictating splicing patterns triggered by the c.386G>A mutation.

### TIA1 splicing factor plays a role in governing specie-specific splicing profiles

**Fig. 3:**
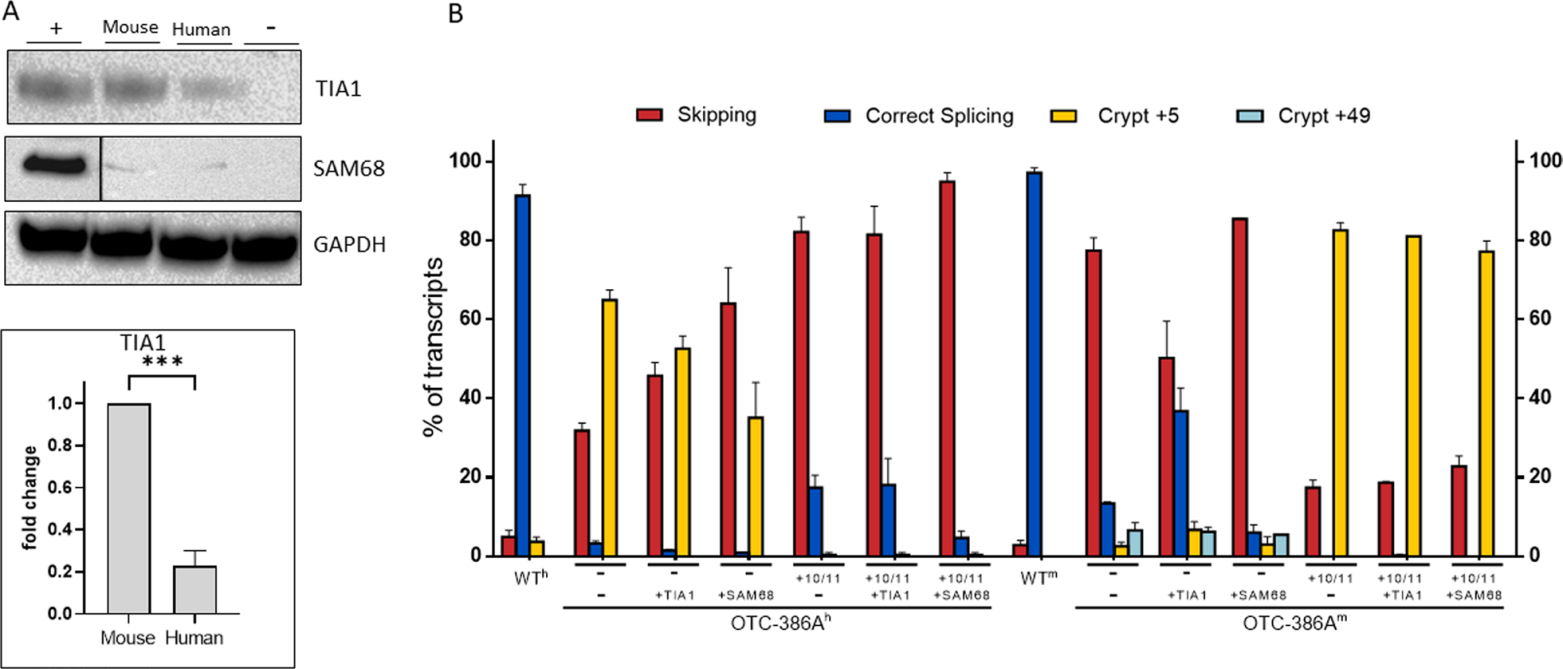
TIA1 preferentially binds nucleotide variations at +10-11 positions in mouse. **A** Western blot analysis of pulldown assays. Results are presented as mean ± standard deviation (SD) of three independent experiments and a representative blot is shown (*NE* nuclear extract input; *C-* naked beads). Histograms report fold changes over the wild-type. *p < 0.1; **p < 0.01; ***p < 0.001; ****p < 0.0001. **B** Splicing patterns of OTC^h^ and OTC^m^ minigenes in Hepa1-6/HepG2 cells expressing minigenes variants alone or in combination with TIA1 or Sam68-expressing plasmids. Bar plots report the relative percentage of each transcript, which is expressed as mean ± standard deviation (SD) from three independent experiments. The transcript type is indicated on the top

The computational prediction of splicing factor binding sites in the exon 4 5′ss (Additional file 3: Fig. S2) suggested a preferential binding of TIA1 and Sam68 to the mouse and human sequences, respectively. Interestingly, swapping +10-11 nucleotides between human and mouse sequences resulted in an opposite prediction. To experimentally validate the bioinformatics analysis, we performed RNA oligonucleotide binding assays. Biotinylated 2-O Me RNA oligonucleotides with mouse or human sequences spanning from position +1 to +20 were incubated with HeLa cell nuclear extract and, after elution, binding of Sam68 and TIA1 analyzed by Western blotting. The results showed negligible binding of Sam68 in both human and mouse context, while TIA1 exhibited preferential binding (4.3×, p = 0.0002) for the mouse sequence compared to the human context (Fig. 3A).

To confirm the involvement of TIA1 in the specie-specific splicing pattern, we performed TIA1 and Sam68 overexpression.

Consistently, overexpression of Sam68 with mutated OTC-386A minigenes was ineffective in both contexts whereas that of TIA1 remarkably increased usage of the authentic 5′ss in the mouse context only (Fig. 3B and Additional file 4: Fig. S3).

These data indicate a functional role of the TIA1 splicing factor in the modulation of species-specific alternative splicing profiles associated with the c.386G>A mutation.

### The interplay between the authentic and the cryptic 5′ss modulates the pathogenic effect of naturally-occurring OTCD-associated mutations

**Fig. 4:**
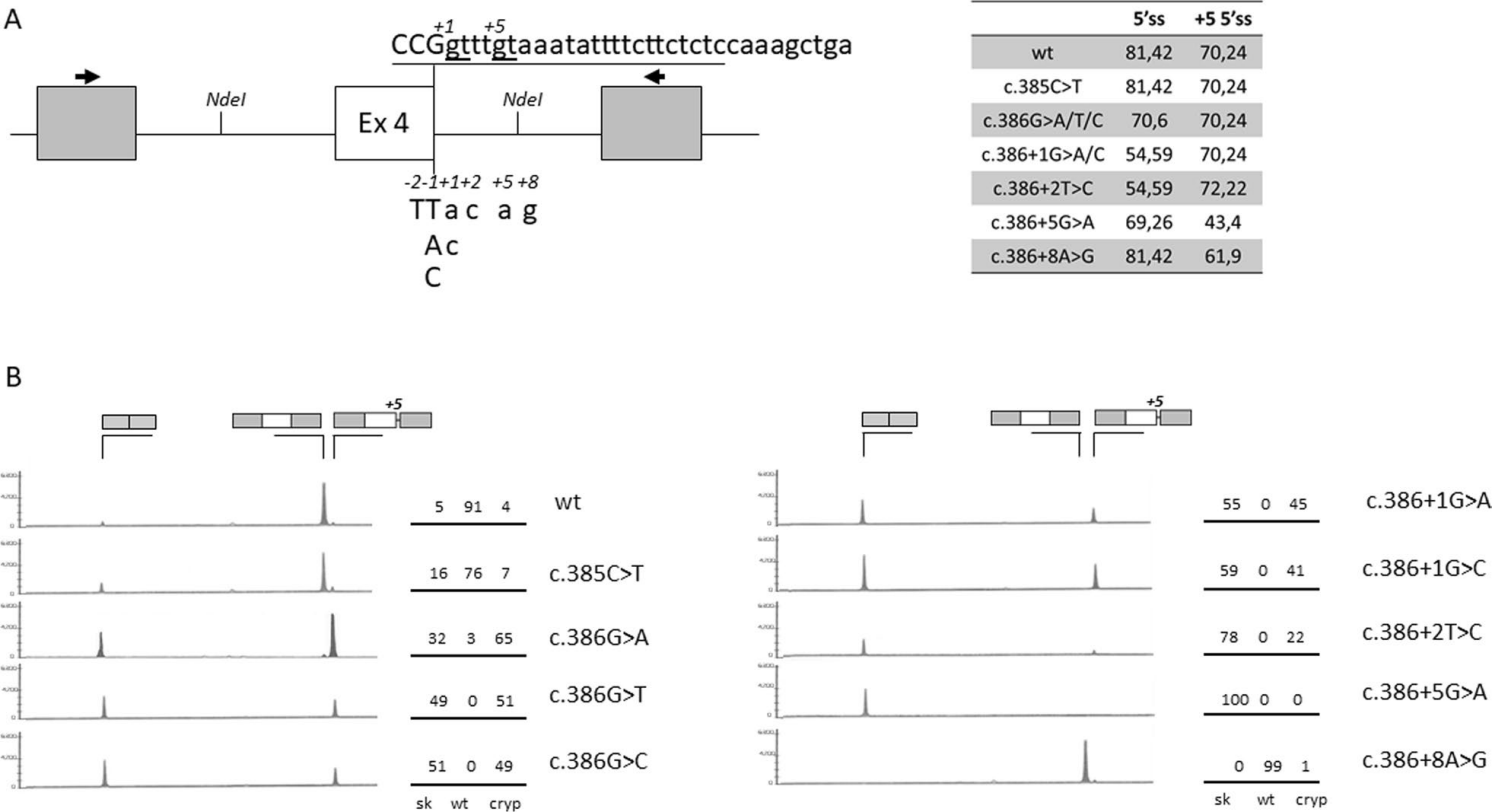
The interplay between the authentic and the cryptic 5′ss governs the pathogenic effect of natural OTC mutations. **A** Schematic representation of the human *OTC* exon 4 genomic sequence cloned as minigene in the pTB vector within the *Nde*I sites. Exonic and intronic sequences are represented by boxes and lines, respectively. Primers (arrows) used to perform the RT-PCR are indicated on top. The sequences, with exonic and intronic nucleotides in upper and lower cases respectively, report (i) the authentic 5′ss with the positions of the investigated changes detailed below, and (ii) the cryptic 5′ss. Both 5′ss are underlined. The table reports the scores of the 5′ss in the normal sequence and in the presence of different OTC nucleotide changes found in OTCD patients. **B** OTC^h^ splicing patterns evaluated by RT-PCR and denaturing capillary electrophoresis on RNA isolated from HepG2 transiently transfected with the wild-type (wt) minigene or harboring the indicated nucleotide changes. The schematic representation of transcripts (with exons not in scale) is reported on top. Numbers on the right report the relative percentage of each transcript type

A panel of nucleotide changes occurring in this region (Fig. 4A), and described in OTCD patients (www.hgmd.cf.ac.uk/ac/index.php; https://databases.lovd.nl/shared/genes/OTC), offered us the opportunity to investigate the interplay between the authentic and the cryptic 5′ss. The bioinformatic analysis of 5′ss strength predicts a differential influence of changes on the two 5′ss (Fig. 4A, right panel), with some that would affect only the authentic one (c.386G>T; c.386G>C; c.386+1G>A; c.386+1G>C; c.386+2T>C) or the cryptic (c.386+8A>G), or both (c.386+5G>A). The variable effect was demonstrated by minigene expression studies (Fig. 4B). In particular the c.385C>T mutation, at −2 position of the 5′ss, had a minor effect on splicing with a slightly decreased proportions of correct transcripts (from 91 to 76%, p = 0.0051) as compared with the wild-type OTC^h^ construct (wt^h^); the substitutions at positions c.386 (−1 of the 5′ss), +1 and +2 led to barely appreciable levels of correct transcripts, together with exon skipping and usage of the cryptic 5′ss; the G>A substitution at position +5, an highly represented mutation at 5′ss annotated in the human mutation database (http://www.hgmd.cf.ac.uk/ac/index.php), is associated with complete exon skipping and no traces of transcript arising from the usage of the cryptic 5′ss, which was abolished by the nucleotide change; the c.386+8A>G change did not alter splicing.

Overall, these data define the molecular bases of OTCD-causing mutations at the 5′ss of *OTC* exon 4 and provide evidence for the interplay between the authentic and the cryptic 5′ss in governing the pathogenic splicing mechanisms.

### The nucleotide context of human and mouse OTC exon 4 accounts for a differential susceptibility to RNA-based correction by engineered U1snRNA

**Fig. 5:**
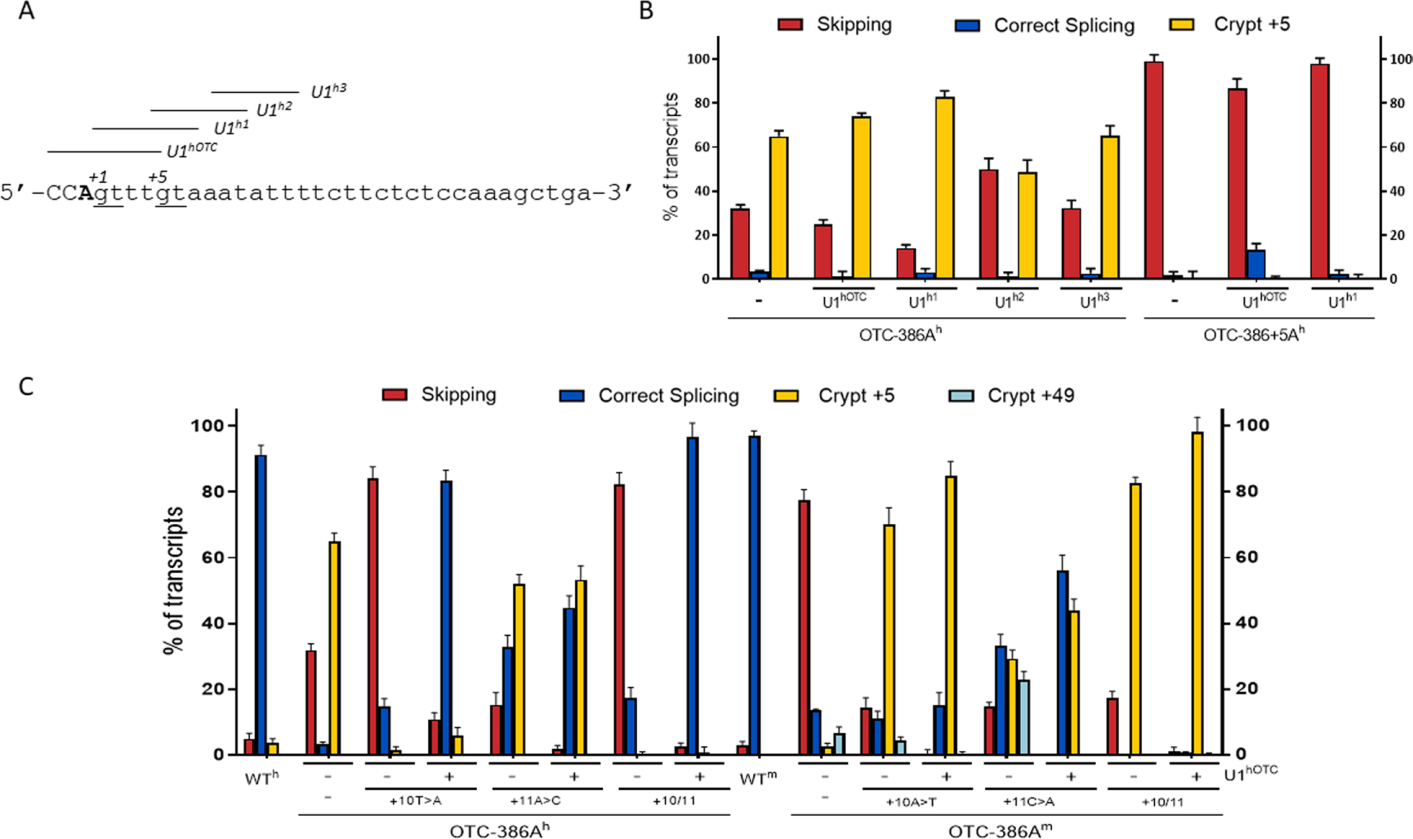
The strong cryptic 5′ss prevents U1snRNA-mediated correction in the human context. **A** Genomic sequence of the human OTC exon 4 exon–intron junction, with exonic and intronic nucleotides in upper and lower cases, respectively. The c.386G>A change is indicated in bold. The regions targeted by modified U1^h^ of the corresponding pre-mRNA are reported on top. **B** Splicing patterns in HepG2 transiently transfected with OTC^h^-386A or OTC^h^-386+5a minigenes alone or in combination with engineered U1^h^, and expressed as bar plots indicating the relative percentage of each transcript. Results are expressed as mean ± SD from three independent experiments. **C** Splicing pattern in HepG2 or Hepa1-6 cells transiently transfected with human and mouse OTC minigenes differing at +10-11 positions alone or in combination with the corresponding U1^hOTC^ designed on the mutated 5′ss. Bar plots indicate the relative percentage of each transcript and results are expressed as mean ± SD from three independent experiments

Based on our previous in vitro and in vivo finding that engineered U1snRNA, either compensatory or exon specific (ExSpe) variants, efficiently rescue splicing of OTC exon 4 in the mouse context (Balestra et al. 2020a), we expressed a panel of U1snRNA variants (Fig. 5A) designed on the human *OTC* context. In co-transfection experiments none of the engineered U1snRNAs appreciably improved selection of the authentic mutated 5′ss in the presence of the c.386G>A mutation but, while decreasing exon skipping, further promoted usage of the adjacent cryptic 5′ss.

The interference of the cryptic 5′ss with the usage of the authentic one was tested by challenging the natural OTC-386+5G>A mutant lacking the cryptic 5′ss. Here, the compensatory U1^hOTC^ appreciably increased the proportion of correct transcripts (from 2 ± 1% to 13 ± 3%, p = 0.0032) (Fig. 5B and Additional file 5: Fig. S4).

Intrigued by the observation on the role of nucleotides at position +10-11 in dictating the preference for the two neighboring 5′ss in the human background, we evaluated the U1snRNA efficacy in the previously created mouse-like OTC-386A^h^ variants. As observed in Fig. 5C and Additional file 6: Fig. S5, changes at nucleotides +10 and +11, either singularly or in combination, rendered the OTC-386A^h^ rescuable by the U1^hOTC^. Vice versa, the introduction of the human nucleotides at positions +10-11 in the OTC-386A^m^ prevented the U1snRNA-mediated correction.

**Fig. 6:**
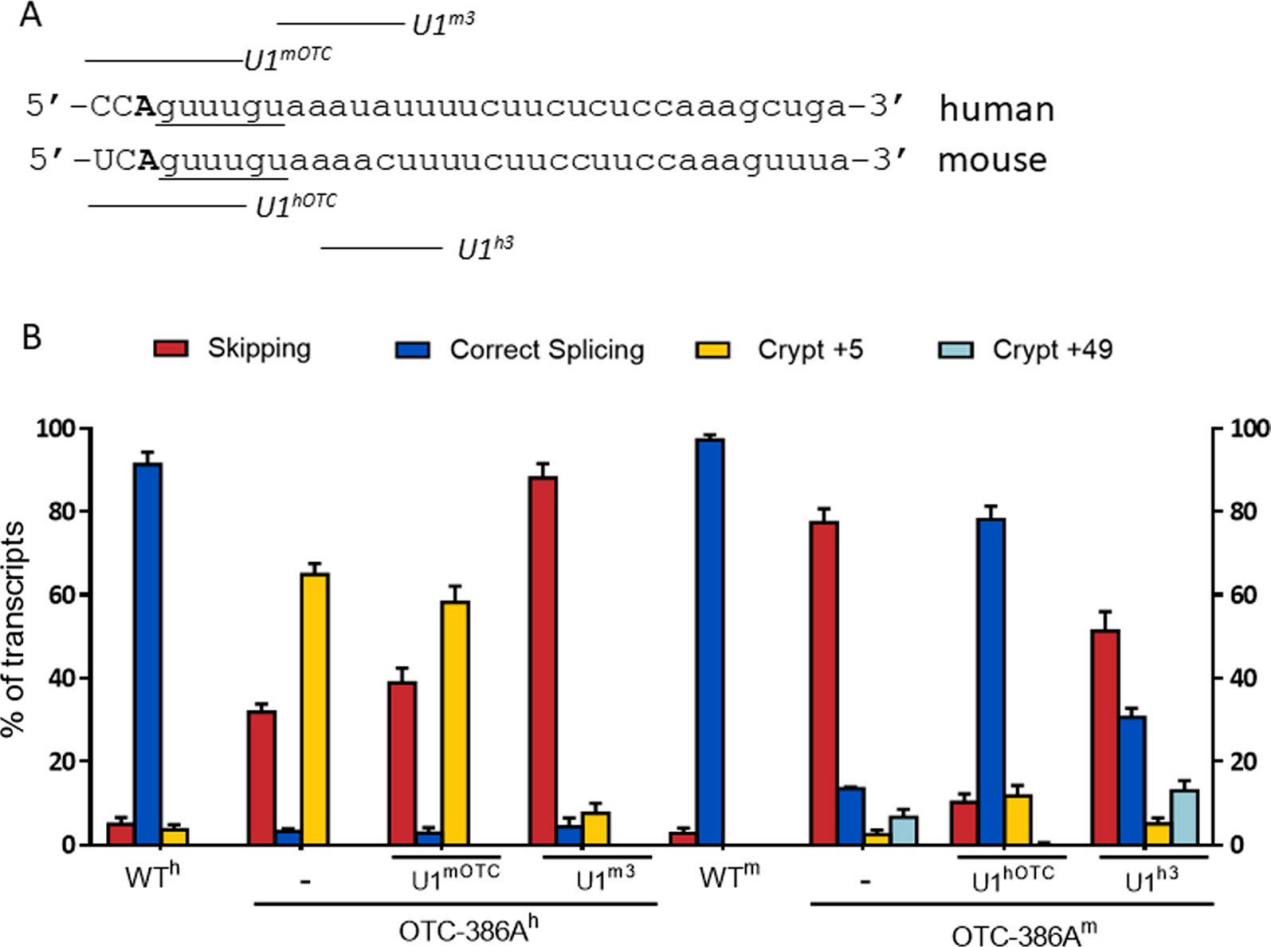
Cross-activity of the human and mouse tailored U1snRNAs. **A** Pre-mRNA sequences of the human and mouse OTC exon 4 exon–intron junctions, with exonic and intronic nucleotides in upper and lower cases, respectively. The regions targeted by the 5′ tail of the modified U1snRNAs are reported (solid line). The c.386G>A change is indicated in bold. **B** The bar plots report the splicing patterns in HepG2 or Hepa1-6 transiently transfected with the OTC-386A^h^ (left) or OTC-386A^m^ (right) minigenes, respectively, and challenged with the U1 variants. Bar plots indicate the relative percentage of each transcript, and results are expressed as mean ± SD from three independent experiments

Due to the high homology between the human and mouse *OTC* exon 4, we also challenged the modified U1snRNAs designed on OTC-386A^h^ towards the OTC-386A^m^, and vice versa (Fig. 6A). In co-transfection assays, the human-tailored U1^hOTC^ appreciably rescued the OTC-386A^m^ to an extent comparable to that obtained with the mouse-tailored U1^mOTC^ and U1^m3^. On the other hand, the mouse-tailored U1snRNAs failed to correct splicing in the OTC-386A^h^ context (Fig. 6B and Additional file 7: Fig. S6).

Overall data demonstrated that the strong proximal cryptic 5′ss in the human *OTC* context prevents U1snRNA-mediated correction of the c.386G>A mutation.

## Discussion

The mouse is the most commonly used animal to model human disease. However, despite the striking genetic homology with human genome that makes studying mice so insightful to understand human diseases, numerous studies showed discrepancies between the human and mouse phenotypes in pathological condition (Elsea and Lucas 2002; Bochner et al. 2013; Telias 2019; Brown et al. 2020).

Aberrant splicing is a common outcome in the presence of exonic or intronic variants that might hamper the intricate network of interactions defining an exon in a specific gene context. Therefore, the evaluation of the functional, and potentially pathological, role of nucleotide changes remains one of the major challenges in the modern genomic era (Taneri et al. 2012; Hartin et al. 2020). This aspect has also to be taken into account during the pre-clinical evaluation of innovative therapeutic approaches in animal models of human diseases. This is of particular relevance when developing therapeutics acting on splicing, an intriguing and expanding research area for several disorders (Donadon et al. 2018, 2019; Yamazaki et al. 2018; Lee et al. 2019; Donegà et al. 2020).

Ornithine TransCarbamylase Deficiency (OTCD) is the most frequent urea cycle disease, an X-linked recessive trait caused by mutations in OTC gene, which is expressed in liver and encodes a mitochondrial enzyme. There is no cure for OTCD but only treatments to limit hyperammonemia, and the disease features make a strong quest for alternative therapies. Among the OTCD mouse models, the mouse spf^ash^ is characterized by the c.386G>A splicing mutation and it has been exploited to evaluate replacement gene therapy approaches (Moscioni et al. 2006; Wang et al. 2011, 2012; Cunningham et al. 2013). This animal model represents therefore an ideal pre-clinical platform to assess the therapeutic potential of RNA therapeutics based on engineered variants of the spliceosomal U1snRNAs, which were proven to be capable, both in cellular (Glaus et al. 2011; Schmid et al. 2013; Scalet et al. 2017, 2018, 2019; Martínez-Pizarro et al. 2018; Balestra et al. 2019a, b; Martín et al. 2021) and animal (Balestra et al. 2014, 2016, 2020b; Dal Mas et al. 2015; Rogalska et al. 2016) models of human diseases, to counteract splicing mutations and force exon recognition, thus rescuing gene expression. Recently, a U1snRNA variant, upon delivery via an Adeno-Associated virus Vector in spf^ash^ mice, partially restored OTC expression at RNA and protein level in liver (Balestra et al. 2020a). On the other hand, translation to humans requires careful evaluation of splicing regulatory elements accounting for species-specific splicing profiles, and potentially affecting responsiveness to splicing-switching molecules.

As a matter of fact, the OTCD-causing c.386G>A mutation leads to different splicing patterns in the human and mouse contexts. By combining computational analysis and minigene assays we demonstrated that, with the exception of the cryptic 5′ss at position +49 of intron 4, which is a peculiar feature of the mouse genomic context, variations at intronic +10-11 positions modulate the interplay between the authentic and the adjacent (+5) cryptic 5′ss, and explain the differential *species-specific* impact of the c.386G>A mutation, and the milder effects in the mouse context. Pull-down and over-expression assays revealed that the TIA1 splicing factor preferentially binds at positions +10-11 in the mouse context, and has a functional role. As previously reported (Förch et al. 2002; Gal-Mark et al. 2009; Wang et al. 2014), TIA1 can favor the recruitment of U1snRNP at the 5′ss through the interaction with the U1snRNP subunits U1C. Therefore, interaction of the endogenous U1snRNP with the splicing factor TIA1, bound in the proximity of the 5′ss of OTC exon 4, would explain the *species-specific* splicing outcome observed in the spf^ash^ mouse model, leading to a better exon definition and thus selection of the authentic but mutated 5′ss. Conversely, the prevalent usage of the adjacent cryptic 5′ss in humans seems the results of the poor interaction with TIA1, disfavoring recognition of the authentic 5′ss by the U1snRNA in the presence of the mutation. In fact, it is well reported that U1snRNA interact with the 5′ss through base-pairing with nucleotides from −3 to +6 positions with reference to the exon–intron junction, extending also up to +7-8 positions (Tan et al. 2016).

These species-specific elements also explained the inability of engineered U1snRNA, either complementary or exon-specific, to re-direct usage of the authentic, albeit mutated, 5′ss in the human context, a correction effect that was restored with the incorporation of the mouse nucleotides at positions +10-11. It is worth noting that the compensatory human-tailored U1^hOTC^ appreciably rescued the OTC-386A^m^ thus ruling out that their ineffectiveness on hOTC was due to inefficient incorporation of the U1snRNA into a functional U1snRNP within cells or to reach the target. Taken together these data demonstrated that, in the h*OTC* context, the strong proximal cryptic 5′ss and the lack of binding of the splicing factor TIA1 helping correct 5′ss definition prevents U1snRNA-mediated correction of the c.386G>A mutation.

On the other hand, the interplay between the authentic and the proximal cryptic 5′ss in hOTC dictates the alternative splicing profile triggered by nucleotide changes occurring in this region and associated with OTCD in patients, and provide insights into their pathogenic role. In particular, the c.385C>T change, occurring at position −2 of the 5′ss, had a minor effect on splicing, a finding that points toward a major pathogenic role of the corresponding amino acid change (p.R129C) on OTC protein. The c.386+8A>G change did not alter splicing, thus leading to classify it as a silent polymorphism. Variants at positions −1 (c.386G>A, c.386G>T, and c.386G>C), +1 (c.386+1G>A, c.386+1G>C) and +2 (c.386+2T>C) led to barely appreciable levels of correct transcripts. Although studies in mice reported that the p.R129H amino acid change does not impair OTC activity (Hodges and Rosenberg 1989), and not excluding a contribution of the underlying p.R129L and p.R129P amino acid changes on OTC protein, these data indicate that these mutations exert their main pathogenic role via splicing impairment. Finally, the c.386+5G>A variant, a highly represented mutation at 5′ss annotated in the human mutation database (http://www.hgmd.cf.ac.uk/ac/index.php), leads to complete exon skipping. Noticeably, this mutation, by abrogating the cryptic 5′ss, was rescued by the U1^hOTC^ variant, thus further strengthening a mechanism where competition between 5′ss is governed by a downstream splicing regulatory element.

## Conclusions

In conclusion, we dissected the molecular mechanisms underlying *species*-specific splicing profiles triggered by the c.386G>A mutation, relatively frequent in humans, and its responsiveness to U1snRNA-mediated correction, and provided the rationale to humanize the spf^ash^ and mimic the OTCD molecular phenotype. Subtle changes accounting for differential binding of splicing factors such as TIA1 at positions +10-11 govern the competition between alternative 5′ss, which in turn can be modulated by naturally-occurring mutations. Data refined the classification and pathogenic mechanisms of human OTC mutations, which assist genetic counselling, and highlight the importance to carefully investigate *specie*-specific molecular mechanisms for translational purposes.

## Supplementary Information

**Additional file 1: Table S1.** Oligonucleotides for creation of minigenes and analysis of splicing. **Table S2.** Oligonucleotides for creation of engineered U1snRNA.

**Additional file 2: Figure S1.** The sequence at intronic positions +10 and +11 explains species-specific splicing patterns. **A** Evaluation by capillary electrophoresis of OTC splicing patterns in HepG2 and Hepa1-6 cells transiently transfected with human and mouse minigenes. The schematic representation of the transcripts is reported on top. **B** The electropherogram reports the sequence of the aberrant transcripts arising from the usage of the alternative 5′ss at position +5. Exon 4 is indicated by the white box.

**Additional file 3: Figure S2.** Bioinformatic analysis of splicing factors show TIA1 preferential binding at nucleotide variations +10-11 in mouse. Bioinformatic analysis of splicing factors binding at mouse (upper) and human (lower) 5′ss of exon 4. Exonic and intronic sequences are indicated in upper and lower case, respectively. Bar plots report the positive and negative scores of target sequences that facilitate exon and intron definition, respectively. Bars have variable width and height respectively related to the number of nucleotides of the binding site and to its score (binding affinity). The label over each bar indicates the name of the protein predicted to bind moreover overlapping bars.

**Additional file 4: Figure S3.** Overexpression of TIA1, but Sam68, remarkably increased usage of the authentic 5′ss in the mouse context. Evaluation by capillary electrophoresis of OTC splicing patterns in HepG2 and Hepa1-6 cells transiently transfected with the human or mouse minigenes alone or in combination with TIA1 or Sam68-expressing plasmids. The schematic representation of the transcripts is reported on top.

**Additional file 5: Figure S4.** The compensatory U1^hOTC^, but not the ExSpe U1^h1^, partially rescue the natural c.386+5G>A mutation. Evaluation by capillary electrophoresis of OTC splicing patterns in HepG2 cells transiently transfected with the human minigenes, harboring the c.386G>A or c.386+5G>A variants, alone or in combination with engineered U1snRNA variants, either the complementary (U1^hOTC^) and Exon Specific (U1^h1^, U1^h2^, U1^h3^) ones. The schematic representation of the transcripts is reported on top.

**Additional file 6: Figure S5.** Nucleotides +10 and +11, either singularly or in combination, renders the human c.386G>A variant rescuable by the compensatory U1^hOTC^. Evaluation by capillary electrophoresis of OTC splicing patterns in HepG2 or Hepa1-6 cells transiently transfected with human and mouse OTC minigenes differing at +10-11 positions alone or in combination with the corresponding U1^hOTC^ designed on the mutated 5′ss. The schematic representation of the transcripts is reported on top.

**Additional file 7: Figure S6.** Cross-activity of the human and mouse tailored U1snRNAs. Evaluation by capillary electrophoresis of OTC splicing patterns in HepG2 or Hepa1-6 cells transiently transfected with the OTC-386A^h^ (left) or OTC-386A^m^ (right) minigenes, respectively, and challenged with the U1 variants. The schematic representation of the transcripts is reported on top.

## Data Availability

All data generated or analysed during this study are included in this published article and its Additional files 1, 2, 3, 4, 5, 6, and 7.

## Abbreviations

5′ss: 5′ Donor splice site
AS: Alternative splicing
ExSpeU1: Exon specific U1snRNA
OTC: Ornithine TransCarbamylase
OTCD: Ornithine TransCarbamylase Deficiency
ss: Splice site
U1snRNA: U1 small nuclear RNA
U1snRNP: Spliceosomal U1 ribonucleoprotein
