# Supplementary material for: OTC intron 4 variations mediate pathogenic splicing patterns caused by the c.386G>A mutation in humans and spfash mice, and govern susceptibility to RNA-based therapies
Source: Mol Med. 2021 Dec 14;27:157. doi: 10.1186/s10020-021-00418-9 (PMC8670272; doi:10.1186/s10020-021-00418-9)
Supplement: Supplementary file 1 — Additional file 1: Table S1. Oligonucleotides for creation of minigenes and analysis of splicing. Table S2. Oligonucleotides for creation of engineered U1snRNA. [file 10020_2021_418_MOESM1_ESM.docx]

S1 Table

| Oligonucleotide |  | Sequence (5’🡪3’) |
| --- | --- | --- |
| Creation of human minigene | | |
| hOTC | Forward | tttaaacatatgcaacccctgcctcccagattcaagcg |
|  | Reverse | tttaaacatatgtctattctccttggtcctggc |
| Analysis of OTC splicing pattern | | |
| Alpha | Forward | caacttcaagctcctaagccactgc |
| Bra | Reverse | taggatccggtcaccaggaagttggttaaatca |
| Mutants for human minigene | | |
| hOTC IVS4 -1G>A/T/C | Forward | cacggacacggccc**h**gtttgtaaatattttc |
|  | Reverse | gaaaatatttacaaac**d**gggccgtgtccgtg |
| hOTC IVS4 +1G>A/T/C | Forward | cacggacacggcccg**h**tttgtaaatattttc |
|  | Reverse | gaaaatatttacaaa**d**cgggccgtgtccgtg |
| hOTC IVS4 +2T>C | Forward | cacggacacggcccgg**c**ttgtaaatattttc |
|  | Reverse | gaaaatatttacaa**g**ccgggccgtgtccgtg |
| hOTC IVS4 +5G>A | Forward | ggacacggcccggttt**a**taaatattttcttctc |
|  | Reverse | gagaagaaaatattta**t**aaaccgggccgtgtcc |
| hOTC IVS4 -2C>T | Forward | cacggacacggcc**t**ggtttgtaaatattttc |
|  | Reverse | gaaaatatttacaaacc**a**ggccgtgtccgtg |
| Mutants for hybrid minigene | | |
| hOTC IVS4 +10 T/A | Forward | gcccagtttgtaaa**a**attttcttctctcc |
|  | Reverse | ggagagaagaaaat**t**tttacaaactgggc |
| mOTC IVS4 +10 A/T | Forward | gctcagtttgtaaa**t**cttttcttccttcc |
|  | Reverse | ggaaggaagaaaag**a**tttacaaactgagc |
| hOTC +11 A/C | Forward | ggcccagtttgtaaat**c**ttttcttctctccaaag |
|  | Reverse | ctttggagagaagaaaa**g**atttacaaactgggcc |
| mOTC +11 C/A | Forward | gacaccgctcagtttgtaaaa**a**ttttcttccttcc |
|  | Reverse | ggaaggaagaaaa**t**ttttacaaactgagcggtgtc |
| hOTC +10-11 TA/AC | Forward | cacggcccagtttgtaaa**ac**ttttcttctctccaaag |
|  | Reverse | ctttggagagaagaaaa**gt**tttacaaactgggccgtg |
| mOTC +10-11 AC/TA | Forward | gacaccgctcagtttgtaaa**ta**ttttcttccttcc |
|  | Reverse | ggaaggaagaaaa**ta**tttacaaactgagcggtgtc |
| Analysis of splicing factors | | |
| Mouse | Forward | guuuguaaaacuuuucuucc |
| Human | Forward | guuuguaaauauuuucuucu |

S2 Table

| Oligonucleotide |  | Sequence (5’🡪3’) |
| --- | --- | --- |
| Modified U1snRNA for human minigene | | |
| U1 hOTCex4 | Forward | aggcccaagatctcatACAAACCGGgcaggggagataccatgatca |
| U1 OTCex4 1-9 | Forward | aggcccaagatctcatTTTACAAACgcaggggagataccatgatca |
| U1hOTC Sh6-14 | Forward | aggcccaagatctcatAGATATTTAgcaggggagataccatgatca |
| U1hOTC Sh11-19 | Forward | aggcccaagatctcatGAAGAAGATgcaggggagataccatgatca |
| U1hOTC Sh-11-3 | Forward | aggcccaagatctcatGGCCGTGTCgcaggggagataccatgatca |
| Modified U1snRNA for mouse minigene | | |
| U1 mOTCex4 | Forward | aggcccaagatctcatACAAACCGAgcaggggagataccatgatca |
| U1 OTCex4 1-9 | Forward | aggcccaagatctcatTTTACAAACgcaggggagataccatgatca |
| U1mOTC Sh5-13 | Forward | aggcccaagatctcatAAGTTTTACgcaggggagataccatgatca |
| U1mOTC Sh9-17 | Forward | aggcccaagatctcatAGAAAAGTTgcaggggagataccatgatca |
| U1mOTC Sh-11-3 | Forward | aggcccaagatctcatAGCGGTGTCgcaggggagataccatgatca |
