# Supplementary figures and images for: OTC intron 4 variations mediate pathogenic splicing patterns caused by the c.386G>A mutation in humans and spfash mice, and govern susceptibility to RNA-based therapies
Source: Mol Med. 2021 Dec 14;27:157. doi: 10.1186/s10020-021-00418-9 (PMC8670272; doi:10.1186/s10020-021-00418-9)

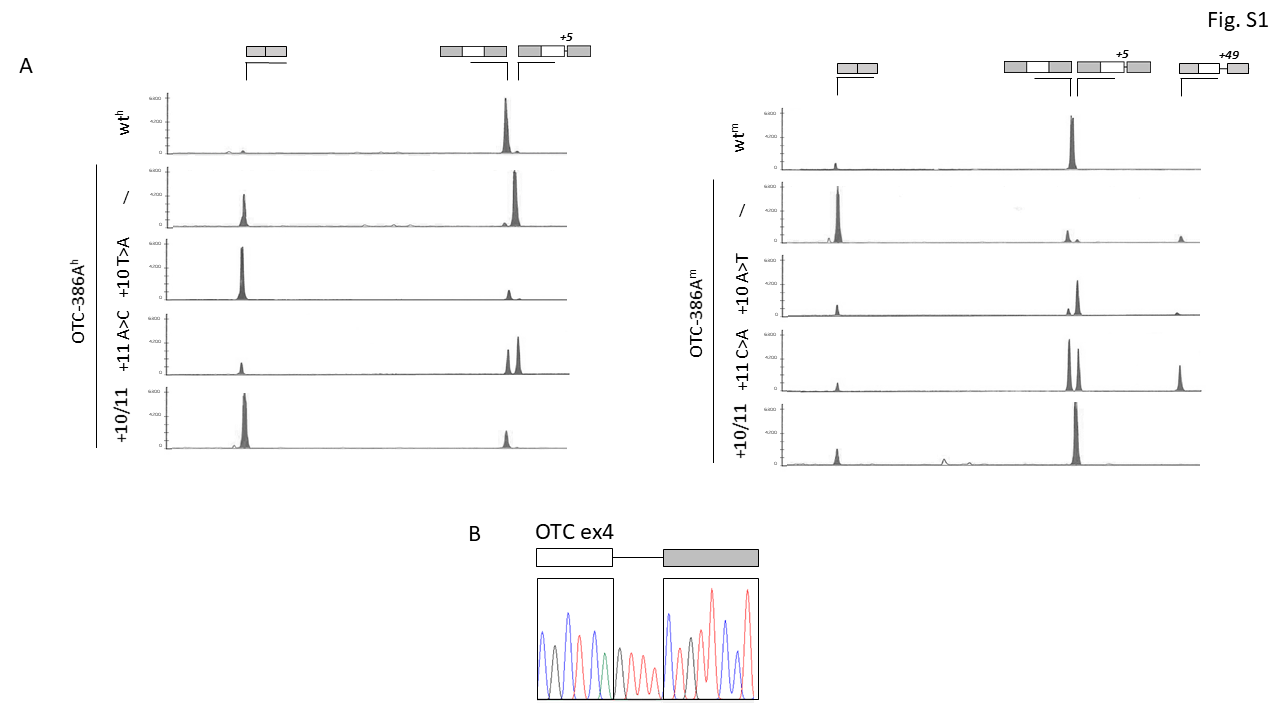

Supplement: Supplementary file 2 — Additional file 2: Figure S1. The sequence at intronic positions +10 and +11 explains species-specific splicing patterns. A Evaluation by capillary electrophoresis of OTC splicing patterns in HepG2 and Hepa1-6 cells transiently transfected with human and mouse minigenes. The schematic representation of the transcripts is reported on top. B The electropherogram reports the sequence of the aberrant transcripts arising from the usage of the alternative 5′ss at position +5. Exon 4 is indicated by the white box. [file 10020_2021_418_MOESM2_ESM.tif]

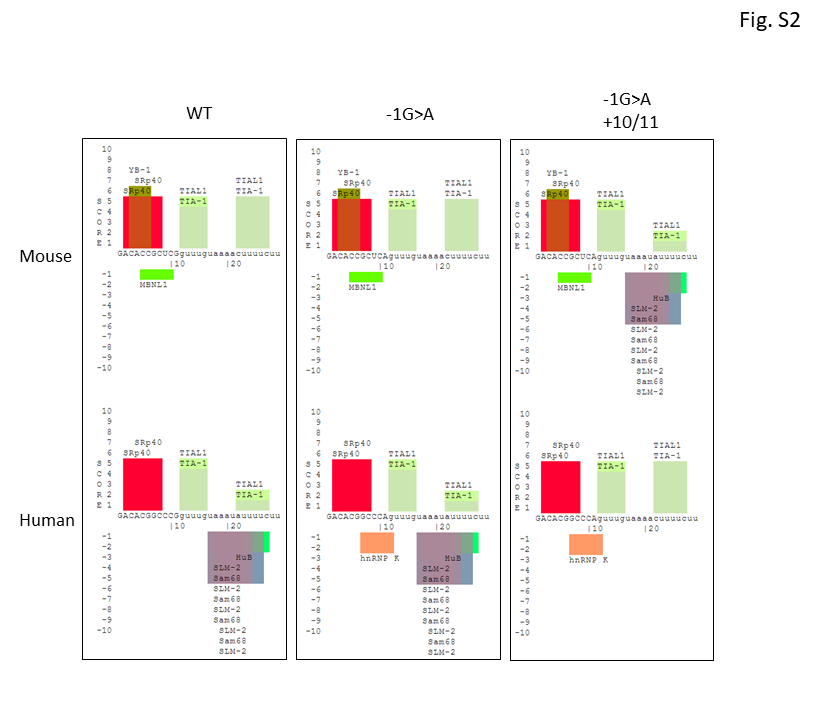

Supplement: Supplementary file 3 — Additional file 3: Figure S2. Bioinformatic analysis of splicing factors show TIA1 preferential binding at nucleotide variations +10-11 in mouse. Bioinformatic analysis of splicing factors binding at mouse (upper) and human (lower) 5′ss of exon 4. Exonic and intronic sequences are indicated in upper and lower case, respectively. Bar plots report the positive and negative scores of target sequences that facilitate exon and intron definition, respectively. Bars have variable width and height respectively related to the number of nucleotides of the binding site and to its score (binding affinity). The label over each bar indicates the name of the protein predicted to bind moreover overlapping bars. [file 10020_2021_418_MOESM3_ESM.tif]

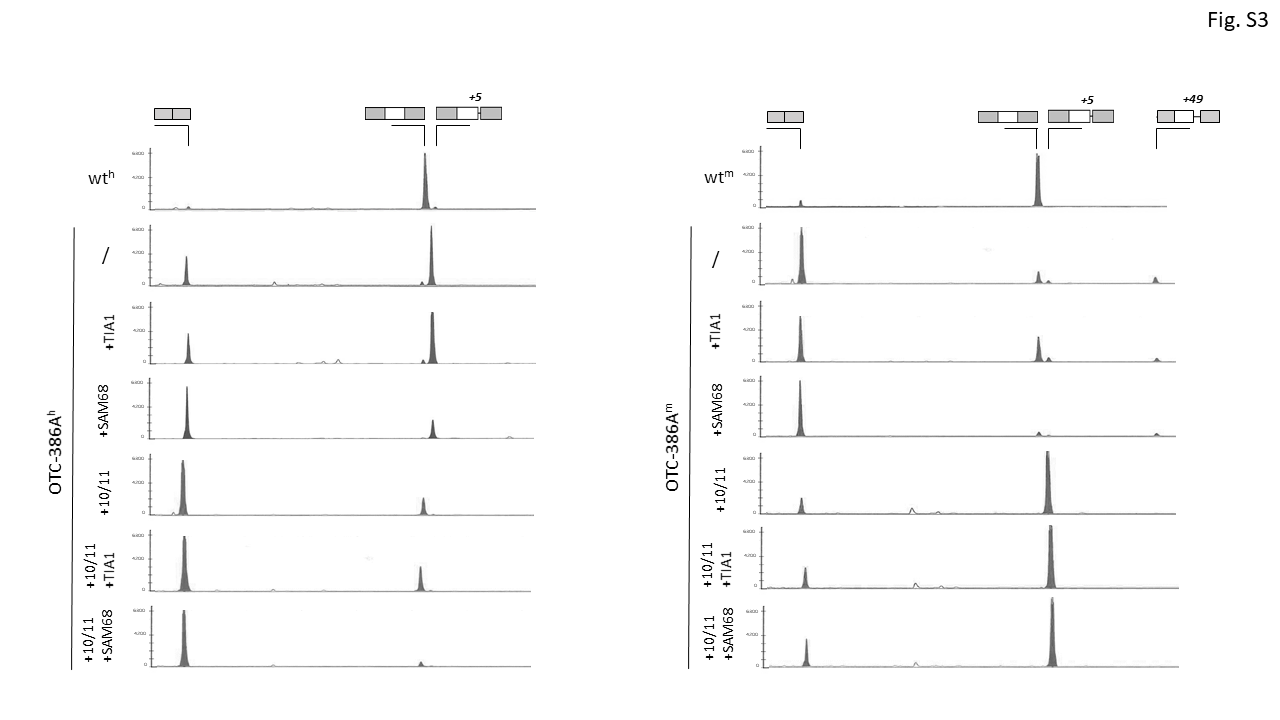

Supplement: Supplementary file 4 — Additional file 4: Figure S3. Overexpression of TIA1, but Sam68, remarkably increased usage of the authentic 5′ss in the mouse context. Evaluation by capillary electrophoresis of OTC splicing patterns in HepG2 and Hepa1-6 cells transiently transfected with the human or mouse minigenes alone or in combination with TIA1 or Sam68-expressing plasmids. The schematic representation of the transcripts is reported on top. [file 10020_2021_418_MOESM4_ESM.tif]

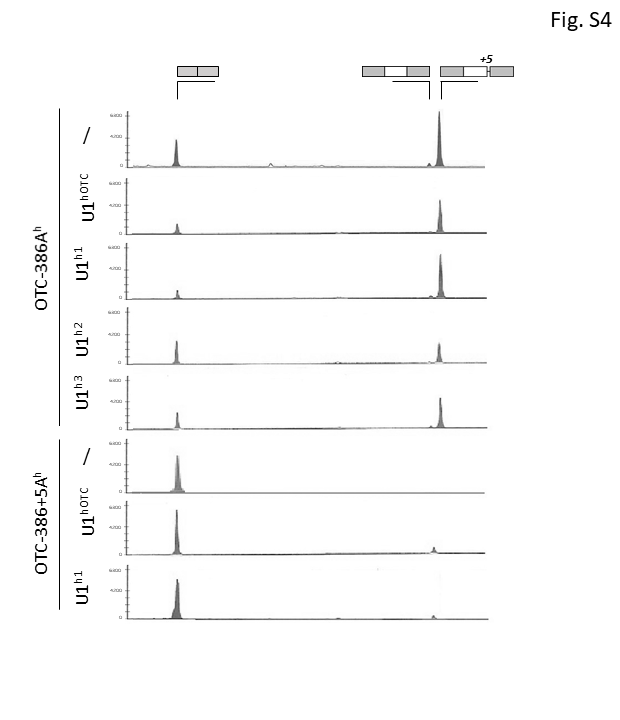

Supplement: Supplementary file 5 — Additional file 5: Figure S4. The compensatory U1hOTC, but not the ExSpe U1h1, partially rescue the natural c.386+5G>A mutation. Evaluation by capillary electrophoresis of OTC splicing patterns in HepG2 cells transiently transfected with the human minigenes, harboring the c.386G>A or c.386+5G>A variants, alone or in combination with engineered U1snRNA variants, either the complementary (U1hOTC) and Exon Specific (U1h1, U1h2, U1h3) ones. The schematic representation of the transcripts is reported on top. [file 10020_2021_418_MOESM5_ESM.tif]

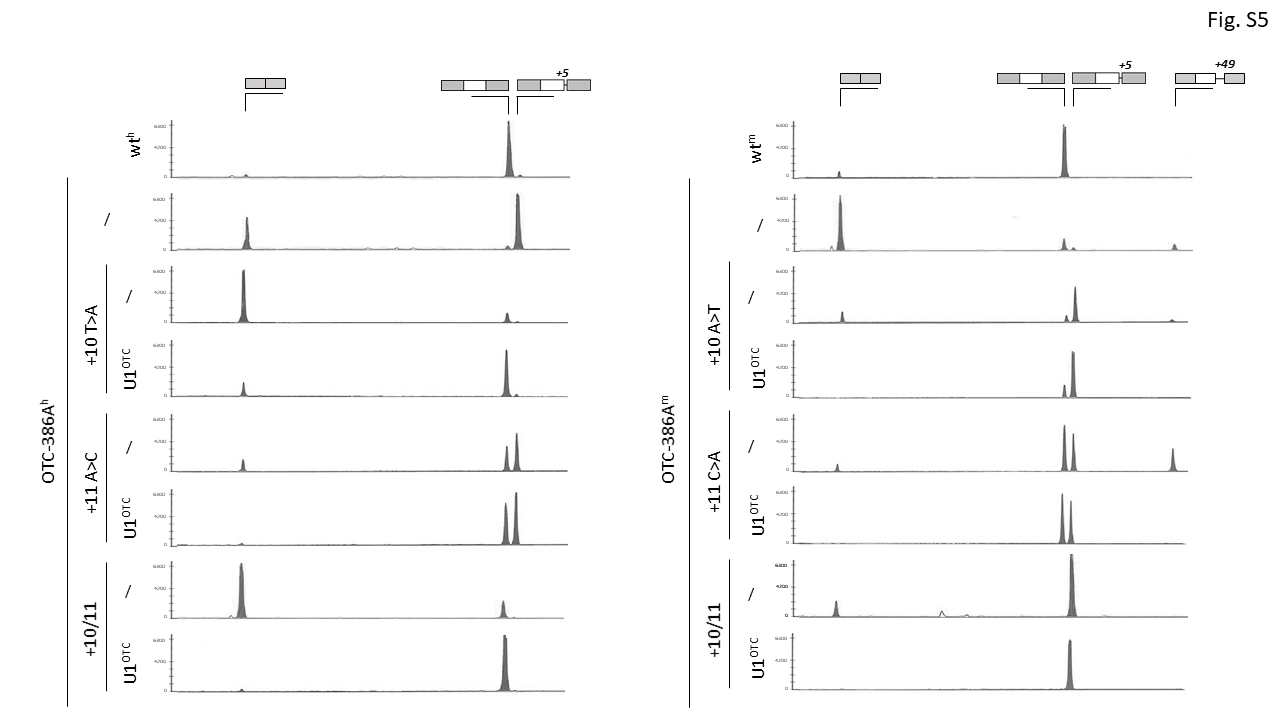

Supplement: Supplementary file 6 — Additional file 6: Figure S5. Nucleotides +10 and +11, either singularly or in combination, renders the human c.386G>A variant rescuable by the compensatory U1hOTC. Evaluation by capillary electrophoresis of OTC splicing patterns in HepG2 or Hepa1-6 cells transiently transfected with human and mouse OTC minigenes differing at +10-11 positions alone or in combination with the corresponding U1hOTC designed on the mutated 5′ss. The schematic representation of the transcripts is reported on top. [file 10020_2021_418_MOESM6_ESM.tif]

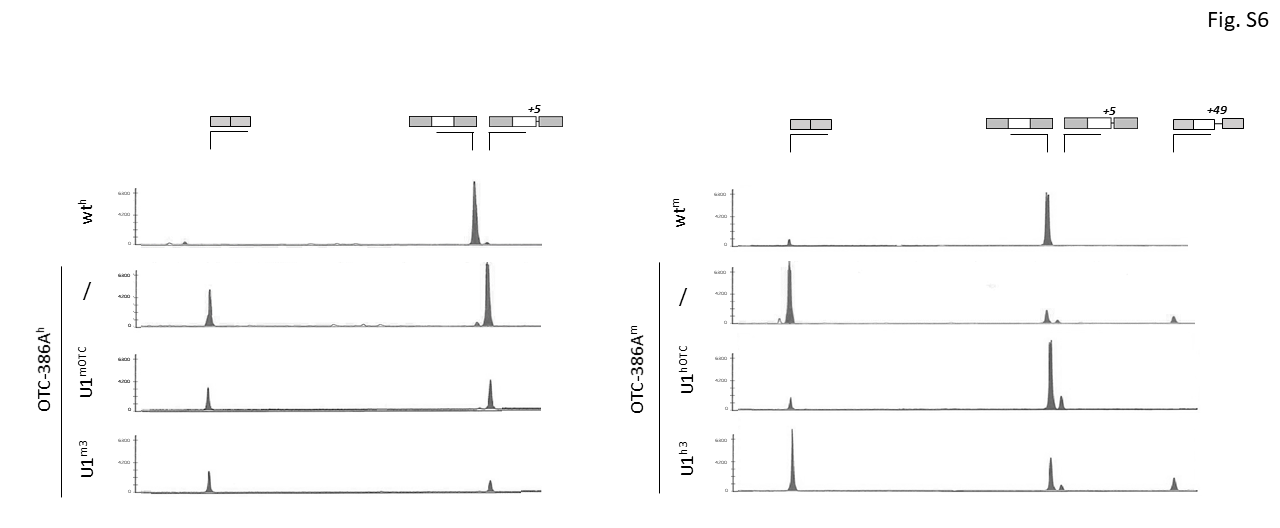

Supplement: Supplementary file 7 — Additional file 7: Figure S6. Cross-activity of the human and mouse tailored U1snRNAs. Evaluation by capillary electrophoresis of OTC splicing patterns in HepG2 or Hepa1-6 cells transiently transfected with the OTC-386Ah (left) or OTC-386Am (right) minigenes, respectively, and challenged with the U1 variants. The schematic representation of the transcripts is reported on top. [file 10020_2021_418_MOESM7_ESM.tif]
